# Supplementary material for: Dysregulation of lncRNA MALAT1 Contributes to Lung Cancer in African Americans by Modulating the Tumor Immune Microenvironment
Source: Cancers (Basel). 2024 May 15;16(10):1876. doi: 10.3390/cancers16101876 (PMC11119359; doi:10.3390/cancers16101876)
Supplement: Supplementary file 1 [file cancers-16-01876-s001.zip › cancers-2979842-supplementary.pdf]

Supplementary Materials

Table S1. Forty-three lung cancer-associated lncRNAs were tested in this study.

No. #	LncRNA	Forward Primer sequence (5'-3')	Reverse Primer sequence (5'-3')
1	AFAP1-AS1	TCGCTCAATGGAGTGACGGCA	CGGCTGAGACCGCTGAGAACTT
2	AGAP2-AS1	TACCTTGACCTTGCTGCTCTC	TGTCCCTTAATGACCCCATCC
3	ANRIL	TTGTGAAGCCCAAGTACTGC	TTCACTGTGGAGACGTTGG
4	ATB	CTTCACCAGCACCCAGAGA	AAGACAGAAAAACAGTTCCGAGTC
5	BANCR	CAAACCTGAATCTCACCTCTGC	TGCCAGGGATGACTTGCGTA
6	BCYRN1	GCCTGTAATCCCAGCTCTCA	GGGTTGTTGCTTTGAGGGAA
7	CAR10	TCTGCTGGACTTAGGCTGGT	TGCTGCAGTGTGTGGCTATC
8	CASC2	GCACATTGGACGGTGTTTCC	CCCAGTCCTTCACAGGTCAC
9	CCAT2	CCCTGGTCAAATTGCTTAACCT	TTATTCGTCCCTCTGTTTTATGGAT
10	DLX6-AS1	ATGTTTGGAGGTTCCCCACC	CGGAAGCCTCGGACCATTTA
11	GAS5	TCCCAGCCTCAGACTCAACA	GTTTCATAGGCCCCTGTGCT
12	H19	GACTCAGGAATCGGCTCTGG	CTGCTGTTCCGATGGTGTCT
13	HMlincRNA717	TGGATGCTTACAAAGGACTGG	CTGCAATTACGGAAAGAGCTG
14	HNF1A-AS1	TCAAGAAATGGTGGCTAT	GCTCTGAGACTGGCTGAA
15	HOTAIR	GGAAAGATCCAAATGGGACCA	CTAGGAATCAGCACGAAGCAAA
16	HOTTIP	CACACTCACATTCGCACACT	TCCAGAACTAAGCCAGCCATA
17	LINC00461	CATTTCCCACCCACAGCCATCT	CTCTTGGCACCCTTTCCACTTG
18	LINC00673	TACCACACCCTTTCTTGCCC	ACACTGGCCTCTTTACACGG
19	LINC01133	GCTGTGGTGGAGAGAATGGA	CCCCAGCTTTCCAGATCCAAA
20	LincRNA-p21	CCCGGGCTTGTCTTTTGTT	GAGTGGGTGGCTCACTCTTCTG
21	lncRNA-LET	AGGAGTCCTTGGACCTGAGC	AGTGGCTGGCATATAACCAACA
22	lncRNA-MVIH	AATTTTGCACATCTGAACAGCC	TTCAAAATCCCACTACGCCCA
23	lnc-TCF7	AGGAGTCCTTGGACCTGAGC	AGTGGCTGGCATATAACCAACA
24	LUADT1	TTTCCGTTCAGCAAATCCACAC	TTAGGTCCAGCACTGTTATCCA
25	MALAT1	CTTCCCTAGGGGATTTCAGG	GCCCACAGGAACAAGTCCTA
26	MEG3	TGTGGGATGTTGGTTTCGCC	CCACATTCGAGGTCCCTTCC
27	MEG8	TGCACTTTGCTGATTGAAGG	TCTCCAGGCTCCATCCTAAA
28	NEAT1	CTTCCTCCCTTTAACTTATCCATTCAC	CTCTTCCTCCACCATTACCAACAATAC
29	PANDAR	TGCACACATTTAACCCGAAG	CCCCAAAGCTACATCTATGACA
30	PCAT-1	TGAGAAGAGAAATCTATTGGAACC	GGTTTGTCTCCGCTGCTTTA
31	PVT1	TGAGAACTGTCCTTACGTGACC	AGAGCACCAAGACTGGCTCT
32	RGMBAS1	AGTGGGCAAACTTCAACGTTC	GAGCTGCCATTGAATTAATCCG
33	RMRP	ACTCCAAAGTCCGCCAAGA	TGCGTAACTAGAGGGAGCTGAC
34	SCAL1	GTGTCAAGCTCGGATTGCCT	GAGCCCACACACTCAGGTTC
35	SNHG1	AGGCTGAAGTTACAGGTC	TTGGCTCCCAGTGTCTTA
36	SNHG11	TGGAAACTGTTAGAGGAAAC	CTCTTGGTCTGTGATCAACC
37	SNHG9	GACTGCAGACCCCTAACCTT	ACCCGCATGCAGTGAGTTA

38	SOX2OT	GCTCGTGGCTTAGGAGATTG	CTGGCAAAGCATGAGGAACT
39	SPRY4-IT1	GCTGAGCTGGTGGTTGAAAGGAATC	GCTTGGCCCACGATGACTTGG
40	TUG1	CTGAAGAAAGGCAACATC	GTAGGCTACTACAGGATTTG
41	UCA1/CUDR	CTCTCCATTGGGTTCAC	GCGGCAGGTCTTAAGAGATGAG
42	XIST	GCATAACTCGGCTTAGGGCT	TCCTCTGCCTGACCTGCTAT
43	ZXF1	CTACCGATGAAGGATGGCTGG	ACCTGTGCAGACCCTAATGTT

Table S2. Mean expression levels of seven lncRNAs in lung cancer patients compared to their cancer-free counterparts.

	All controls	All lung cancer	P	AA Controls	AA cancer	P	WA	WA cancer	P
H19	1.601	2.281	0.001	1.730	2.302	0.035	1.481	2.266	0.006
NEAT1	0.842	1.135	0.002	0.902	1.182	0.037	0.789	1.101	0.022
SNHG1	0.827	1.103	0.002	0.837	1.118	0.023	0.817	1.093	0,031
TUG1	0.710	0.987	0.001	0.640	0.862	0.046	0.773	1.079	0.005
MALAT1	0.202	0.271	0.062	0.171	0.338	0.007*	0.208	0.222	0.737
PVT1	0.811	0.891	0.248	0.789	1.059	0.015*	0.830	0.767	0.451
RMRP	1.301	1.523	0.2643	1.500	1.179	0.284	1.122	1.755	0.017

*, p<0.05. The Mann-Whitney U test was used to compare the cytokines between these groups, with an alpha level of 0.05 set as the threshold for determining statistical significance.

Table S3. Associations between the ncRNAs and clinical and demographic data, analyzed using Pearson's correlation coefficients.

	MALAT1	RMRP	H19	NEAT1	PVT1	SNHG1	TUG1	Age	Sex	Pack-smoking years	Types of cancer	Stage	Race
MALAT1		0.653	0.496	0.991	0.065	-0.050	0.082	0.139	-0.032	0.019	-0.165	0.015	0.015
RMRP			0.172	0.858	0.900	0.058	0.062	0.052	0.127	0.027	-0.162	0.013	0.013
H19				0.362	0.787	-0.039	0.152	0.060	-0.042	0.200	0.179	0.173	-0.157
NEAT1					0.467	0.159	0.209	0.005	-0.084	0.021	0.094	-0.011	-0.005
PVT1						0.522	0.632	-0.085	-0.218	0.141	-0.148	-0.077	0.002
SNHG1							0.105	0.105	-0.131	0.016	0.140	0.026	0.137
TUG1								0.074	0.086	0.136	0.233	-0.150	0.066

Table S4. Cytokine levels in plasma of AA and WA lung cancer patients and their control groups.

	AA Controls	AA cancer patients	P	WA Controls	WA cancer patients	P
IL-6	0.3334	0.3854	0.2427	0.3862	0.5755	0.003
IL-8	0.2312	0.8039	<0.0001	0.3617	0.6968	0,0003
IL-10	0.8694	1.198	0.0305	0.8703	0.8635	0.9415
MCP-1	7.18	15.55	0.0002	8.109	7.679	0.6962
TNF-α	0.5014	0.774	0.0131	0.5201	0.8663	0.001
IFN-γ	1.028	1.901	0.0017	0.9837	1.618	0.0032

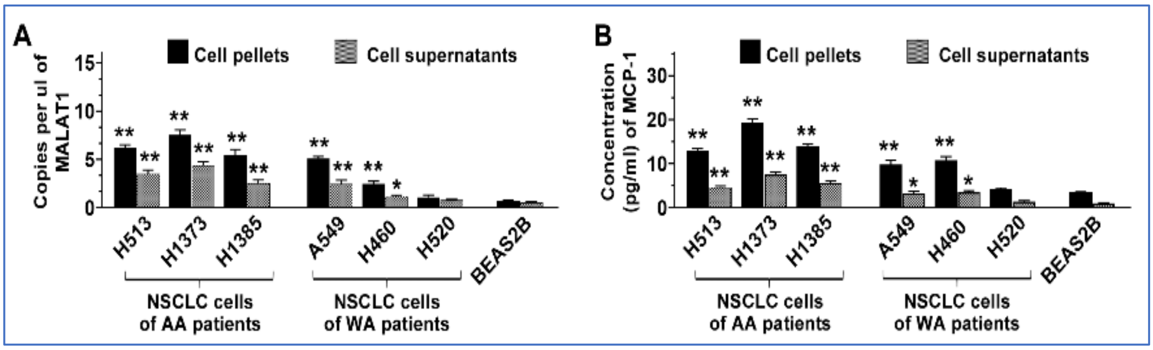

Fig. S1. MALAT1 and MCP-1 are expressed in cancer cell lines. A. This bar chart shows the quantification o MALAT1 in NSCLC cell lines, with individual bars representing cellular pellet and supernatant samples. The MALAT1 levels in NSCLC cell lines, specifically in H513, H1373, A549, H460, and H1385, are elevated in both cell: and supernatants compared to normal cells (BEAS2B), with significant differences observed (*p<0.05; **p<0.01). B. MCP 1 levels in NSCLC cell lines, particularly in H513, H1373, A549, H460, and H1385, are significantly higher in both cell: and supernatants compared to normal cells (BEAS2B), indicating a notable increase (*p<0.05; **p<0.01).

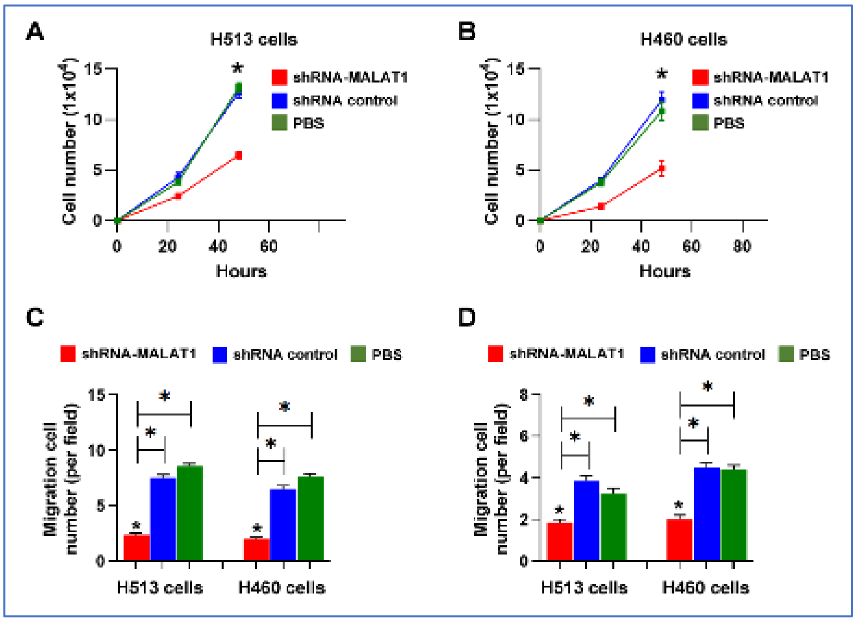

Fig. S2. Effects of MALAT1 knockdown on the proliferation and migration of H513 and H460 cancer cells.

A. Proliferation of H513 cells over time. The graph shows the cell number on the y-axis against time in hours on the x-axis. Three groups are compared: cells treated with shRNA-MALAT1, shRNA control, and PBS. The shRNA-MALAT1 treatment significantly reduced the proliferation rate compared to the control and PBS groups, as indicated by the lower cell counts at each time point. *, all p<0.05. B. Proliferation of H460 cells over time. Similar to panel A, cell numbers are plotted against time for the three groups: shRNA-MALAT1, shRNA control, and PBS. The shRNA-MALAT1 treatment group shows a marked decrease in cell proliferation in

comparison to the control and PBS treatments. *, all p<0.05. C-D. Migration assay shows a significant reduction in cell migration compared to the shRNA control and PBS groups for both H513 and H460 cells. Error bars represent the standard deviation of the mean from three experiments. *, all p<0.05.

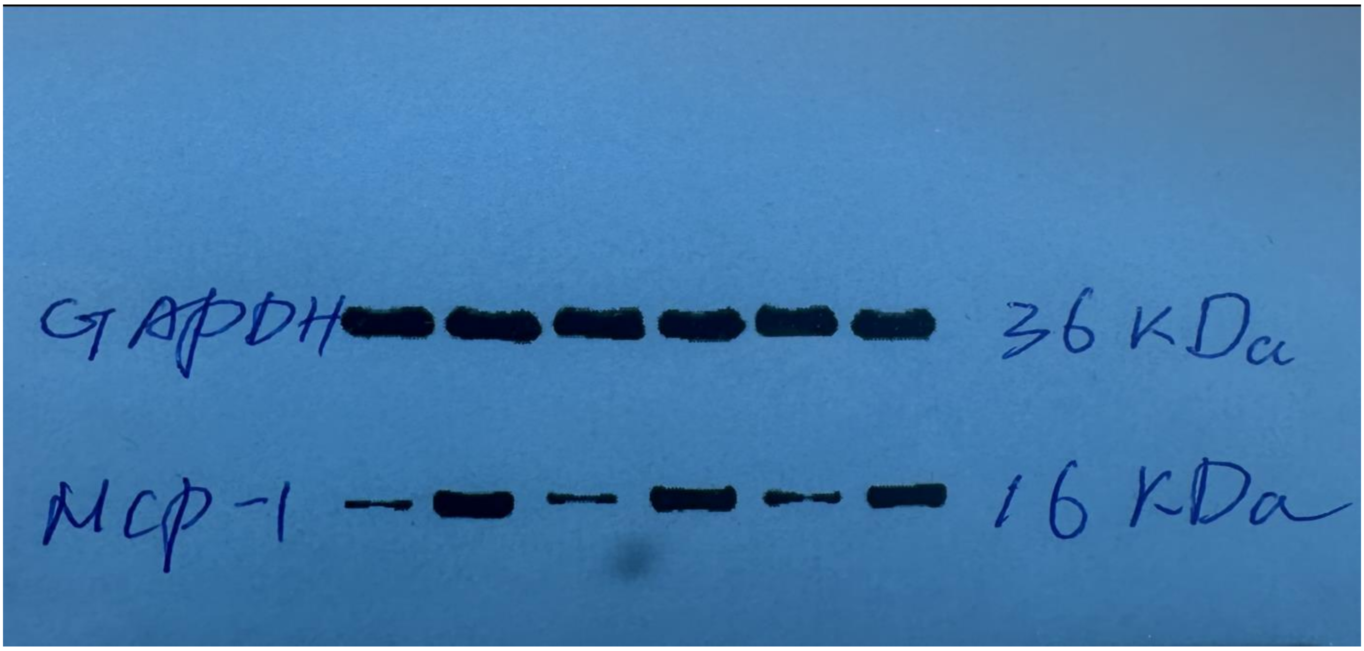

Fig. S3. The uncropped blots correspond to Fig. 5D. For more details, please refer to the legend of Fig. 5.

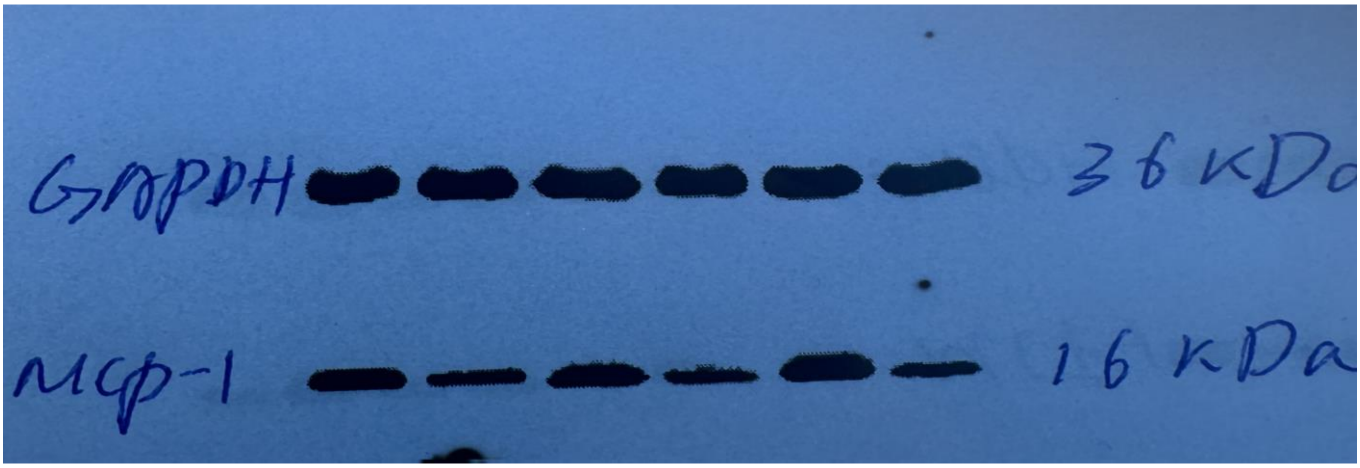

Fig. S4. The uncropped blots correspond to Fig. 5E. For more details, please refer to the legend of Fig. 5.

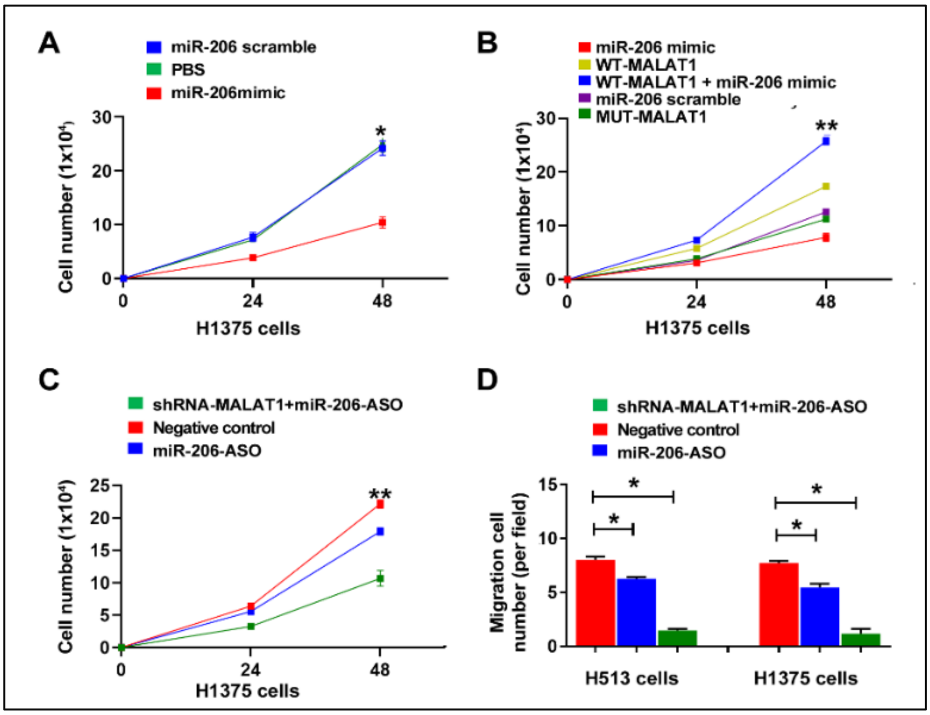

Fig. S5. MALAT1 directly targets miR-206, influencing MCP-1 expression, and contributes to the lung tumorigenesis.

A. Proliferation assay showing cell numbers of lung cancer cells (H1385) treated with either miR-206 mimic or miR-206 scramble control over a 48-hour period. *, $p<0.01$. B. Cell proliferation assays for H1385 cells treated with miR-206 mimic, WT-MALAT1, WT-MALAT1 plus miR-206 mimic, miR-206 scramble, or MUT-MALAT1 over 48 hours. *, all $p<0.05$; **, all $p<0.01$. C. Proliferation assay of H1385 cells treated with sh-MALAT1 plus miR-206-ASO, negative control, or miR-206-ASO alone, showing restored proliferation with miR-206 ASO treatment. *, all $p<0.05$; **, all $p<0.01$. D. Migration assay for H513 and H1385 cells demonstrates the reversion of sh-MALAT1 tumor-suppressive effects by miR-206 ASO. *, all $p<0.05$.
